# Oxidative Stress in Diabetic Nephropathy with Early Chronic Kidney Disease

**DOI:** 10.1155/2016/7047238

**Published:** 2016-07-20

**Authors:** Alejandra Guillermina Miranda-Díaz, Leonardo Pazarín-Villaseñor, Francisco Gerardo Yanowsky-Escatell, Jorge Andrade-Sierra

**Affiliations:** ^1^Department of Physiology, University Health Sciences Centre (Centro Universitario de Ciencias de la Salud), University of Guadalajara, 44150 Guadalajara, JAL, Mexico; ^2^Nephrology Service, Civil Hospital of Guadalajara “Dr. Juan I. Menchaca”, Guadalajara, JAL, Mexico

## Abstract

The increase in the prevalence of diabetes mellitus (DM) and the secondary kidney damage produces diabetic nephropathy (DN). Early nephropathy is defined as the presence of microalbuminuria (30–300 mg/day), including normal glomerular filtration rate (GFR) or a mildly decreased GFR (60–89 mL/min/1.73 m^2^), with or without overt nephropathy. The earliest change caused by DN is hyperfiltration with proteinuria. The acceptable excretion rate of albumin in urine is <30 mg/day. Albuminuria represents the excretion of >300 mg/day. Chronic kidney disease (CKD) is characterized by abnormalities in renal function that persist for >3 months with health implications. Alterations in the redox state in DN are caused by the persistent state of hyperglycemia and the increase in advanced glycation end products (AGEs) with ability to affect the renin-angiotensin system and the transforming growth factor-beta (TGF-*β*), producing chronic inflammation and glomerular and tubular hypertrophy and favoring the appearance of oxidative stress. In DN imbalance between prooxidant/antioxidant processes exists with an increase in reactive oxygen species (ROS). The overproduction of ROS diminishes expression of the antioxidant enzymes (manganese superoxide dismutase, glutathione peroxidase, and catalase). The early detection of CKD secondary to DN and the timely identification of patients would permit decreasing its impact on health.

## 1. Introduction

Diabetes mellitus (DM) is a metabolic disturbance that is characterized by dysfunction in the secretion and response to insulin. The International Diabetes Federation (IDF) predicts that the prevalence of DM will increase from 285 million persons affected in 2010 to 439 million in 2030 (an increase of ~50%). In 2009, it was reported that diabetic nephropathy (DN) causes ~44% of all cases of chronic end-stage renal disease (ESRD) in the United States [1]. Diabetes mellitus contributes, in large part, to the high costs of health care and the increase in mortality from the increased incidence of DN that leads to ESRD, because patients must be inscribed in a program of renal replacement therapy (RRT), whether it be dialysis or kidney transplant [2]. In order for DN to occur several factors should coincide, among which are the effect of genetic susceptibility, hyperglycemia, activation of the polyol pathways, activation of the renin-angiotensin system, activation of the protein kinase C pathway, increase in the advanced glycation end products (AGEs), glomerular hyperfiltration, and the production of reactive oxygen species (ROS) [3].

The first histological changes in DN are considered to be detectable two years after the diagnosis of DM [4]. The DN remains asymptomatic until later stages when the intervention makes it impossible to detain progression of the illness. Therefore, it is urgently necessary to detect the illness before the appearance of complications secondary to DM [5].

## 2. Early Kidney Changes

Normal kidney function is characterized as a glomerular filtration rate (GFR) of ≥90 mL/min/1.73 m^2^ without albuminuria. The GFR can be evaluated using the equations of the Chronic Kidney Disease Epidemiology Collaboration (CKD-EPI) or the formula of the Modification on Diet in Renal Disease (MDRD) Study [7, 8]. As such, early nephropathy is defined by the presence of microalbuminuria (30–300 mg/day), including a normal or mildly diminished GFR (60–89 mL/min/1.73 m^2^), with or without manifested nephropathy [9].

Chronic kidney disease (CKD) is defined as abnormalities in the kidney structure or function for >3 months accompanied by structural damage as evidenced by histopathological studies or images, with health implications [8]. The ESRD is classified according to the guidelines of the initiative of results of quality in kidney disease (National Kidney Foundation, KDOQI Guidelines) [10]. The GFR is one of the most utilized markers for the diagnosis and follow-up of CKD and should be calculated in all patients with DM, based on evaluation in 5 categories of GFR function: G1, GFR ≥90 mL/min/1.73 m^2^; G2, with 60–89 mL/min/1.73 m^2^; G3, a stage that is divided into G3a with GFR from 45–59 mL/min/1.73 m^2^ and G3b with 30–44 mL/min/1.73 m^2^; G4, corresponding to 15–29 mL/min/1.73 m^2^; and G5, known as ESRD established by <15 mL/min/1.73 m^2^ [11] (Table 1(a)). The stages of G1 and G2 are considered early CKD; G3a and G3b are considered overt nephropathy [8, 9, 12], while G4 and G5 are considered advanced stages [13, 14].

The earliest changes caused by DN, even before alterations to the serum creatinine, are due to the hemodynamic changes of glomerular hypoperfusion with an initial increase in GFR known as hyperfiltration [9] which favors albumin leakage from the glomerular capillaries, causing the appearance of proteins in the urine [15]. In fact, the discovery of urinary albumin is the recommended method of detection because it manifests in the majority of nephropathies of chronic evolution and directly affects the sensitivity of the glomerular permeability [16]. Microalbuminuria corresponds to alterations in the excretion of albumin secondary to changes that occur in the glomerulus [17].

## 3. Albuminuria

Albuminuria (including the detection of very low levels) and the albumin-creatinine ratio (A/Cr of 3.5 mg/g) are both considered established risk factors for the progression of cardiovascular disease (CVD) (cerebrovascular accident or acute myocardial infarction) [8]. In healthy people, the acceptable excretion rate of albumin in urine is <30 mg/day, a figure that corresponds to the albuminuria stage A1 [11]. In current clinical practice, it is recommended to consider the stage as A2 when there is a moderate increase of microalbuminuria [11]. The lowest variant of albuminuria in A2 stage is defined as the albumin excretion rate of 20 mcg/min, which is equivalent to 30 mg/day or 30 mg/g of the A/Cr. The highest variant considered in A2 stage is the excretion of 200 mcg/min, equivalent to 300 mg/day and A/Cr of 300 mg/g. Consequently, the microalbuminuria range between 30 and 300 mg/day must be considered for stage A2, as well as in the 24 h collection of urine or the A/Cr. It is estimated that A2 stage progresses to stage A3 ~ 2-3% per year and is associated with a decrease in GFR. It is important to consider A2 stage as a risk factor for progression of the illness to stage A3, although not all patients progress and some even return to the A1 stage. However, it is fundamental to monitor levels of albuminuria since DN can progress relatively quickly to CKD [10]. If the albuminuria is >300 mg/day or the A/Cr is 300 mg/g, it is classified as stage A3 and represents a severe increase in the excretion of urinary albumin [18] (Table 1(b)).

The prevalence of microalbuminuria in the Mexican population with DM is about 40–50%, including all of the transient causes. However, if you exclude those, the persistence of microalbuminuria in the Mexican population is positive in a relatively small sample [9, 19]. It should also be mentioned that macroalbuminuria in the Hispanic population with DM is similar to that reported in the non-Hispanic population [20].

There are some tests available which are relatively low in cost for the early diagnosis of moderate albuminuria. The 24 h urine collection is widely considered the gold standard in diagnostic testing. This test should be confirmed by two additional samples collected over the course of 3–6 months, since the positive diagnosis of stage A2 is determined by 2-3 abnormal tests. It should also be noted that the 24 h urine collection can be tricky and is, above all, susceptible to errors and should not be used when conditions exist which might increase the urinary excretion of albumin, such as in urinary tract infections, or when there is hematuria and other kidney alterations [21]. However, the urinary proteinuria/urinary creatinine ratio, defined as the concentration of proteins in urine divided by the creatinine concentration in a randomly collected urine sample, has been proposed as an alternative to the 24 h urine collection [22], with adequate correlation to the latter test [23].

## 4. Diabetic Nephropathy

Diabetic nephropathy is characterized by albuminuria (>300 mg/day) and a reduced GFR [24]. It should be considered that the albuminuria is sometimes present at the moment when DM is diagnosed, after the kidney has been exposed to chronic hyperglycemia since the prediabetic phase. The mechanisms implicated in the pathogenesis of DN are multiple and complex. The first hemodynamic changes of glomerular hypoperfusion and hyperfiltration favor the leakage of albumin from the glomerular capillaries. Also, structural changes are produced as being characterized by thickening of the glomerular basement membrane, glomerulosclerosis, and expansion of the mesangial cells that lead to kidney fibrosis [25]. Even when the clinical manifestations of DN include diminished GFR and increased levels of urinary excretion of albumin, a substantial proportion of patients with DM have low GFR without albuminuria [26]. Fortunately, only one-third of patients with DM develop nephropathy [27]. However, poor glucose control, arterial hypertension, the increase in cholesterol, and the activation of mediators of inflammation and oxidative stress favor the progress of nephropathy to advanced stages [11]. In fact, recent studies suggest that patients with DM can begin to show signs of kidney disease before the diagnosis of albuminuria [28].

## 5. Triggers of Diabetic Nephropathy

### 5.1. Hyperglycemia

Hyperglycemia seems to be the driving force for the progressive destruction of the glomeruli. The chronically elevated levels of blood glucose lead to the formation of AGEs with posterior hyperfiltration (potential GFR increase of 5–10%) causing glomerular hypertrophy [29]. Hyperglycemia also produces mechanical tension and frictional force which occur in tandem as a result of the hemodynamic changes to the glomeruli, which leads to the liberation of numerous cytokines, proinflammatory markers, and growth factors which stimulate various pathways of oxidative stress [10]. When the DN progresses to later stages, there will be a frank decrease in GFR [30]. Therefore, DN remains substantially underdiagnosed and insufficiently treated [15]. The glycated hemoglobin A1c (HbA1c) was added to the standards of control by the American Diabetes Association (ADA) as a marker of the presence or severity of hyperglycemia in DM, since it exhibits less biological variability than glucose levels and it responds effectively to diet and treatment [31].

## 6. Genetic Factors in Diabetic Nephropathy

Family histories of nephropathy without hypertension are more frequent in patients with nephropathy than in patients with normal function. This suggests the important role of genetic and family factors involved in the development of nephropathy in some patients with type 2 DM [30].

## 7. Arterial Hypertension

Management of arterial pressure is fundamental in patients with CVD to slow the progression of atherosclerotic changes [23]. Diabetes mellitus increases the risk of CVD 2-3 times with the increase in systolic arterial pressure [32]. Arterial hypertension seems to play an important role in DN, since the higher the systolic arterial pressure and the longer the duration of the hypertension, the more serious the nephropathy. It is recommended to control the blood pressure in ranges <140/90 mmHg in all patients with DM, although it is known that 30–50% of patients with DM concomitantly suffer from arterial hypertension [33]. Until recently, the ADA guidelines focused on the objective of achieving systolic arterial pressure of <130 mmHg and diastolic arterial pressure of <80 mmHg in order to decrease proteinuria and slow progression of the DN [10]. The inhibitors of the angiotensin converting enzyme (ACE) or the angiotensin II receptor blockers (ARB) are considered the first line in the management and prevention of the appearance of albuminuria. The use of ACE inhibitors and ARBs in the management of DN seems to prevent progression of the CKD [12]. The ACE inhibitors, like the ARBs, reduce arterial pressure, decreasing the vasoconstrictor effect of the angiotensin II and reducing fibrous infiltration and inflammatory processes in the glomeruli, blocking other secondary signaling pathways. However, the dual therapy of both medications is not recommended in the same patient [34]. It is worth mentioning that the arterial pressure seems to be reasonably well controlled in patients who attend centers of primary medical care [20]. Among the medications initially used by the family doctor is Captopril. As an antecedent, this medication was initially studied in insulin dependent patients. Its administration resulted in a 50% reduction in final results of death, dialysis, and transplant, with the additional benefit of protecting the glomerular filtration [35]. Losartan has been studied in patients with type 2 DM, since it favors a reduction in the incidence of increase in creatinine and the decrease in proteinuria in 35% compared to the conventional antihypertensive treatment [36]. Telmisartan and Enalapril are equivalent in offering significant kidney protection [37].

## 8. Oxidative Stress in Diabetic Nephropathy

Oxidative stress results from the link with the majority of molecular events that underline the pathological process in DN. It is related to alterations in the redox state caused by the persistent hyperglycemic state and the increase in AGEs. These events affect the renin-angiotensin system and the signaling of the transforming growth factor-beta (TGF-*β*), producing chronic inflammation and glomerular and tubular hypertrophy. The renal fibrosis is due primarily to the accumulation of the mesangial cells, favoring the depositing of extracellular matrix (ECM), the thickening of the tubular and glomerular membranes, the dysfunction of podocytes, and the appearance of apoptosis. All of these events are redox alterations that lead to the appearance of albuminuria, proteinuria, glomerulosclerosis, and tubule-interstitial fibrosis [38]. When the redox equilibrium is slightly altered, be it through prolonged increase in the production of ROS or through inefficient antioxidant mechanisms, it can give way to pathological processes. The slight increase in ROS above the physiological limit can induce significant conformational changes in the lipids, proteins, carbohydrates, and nucleic acids, which leads to distorted interactions of the cellular functions. Oxidative stress in DN has the ability to act as a trigger, modulator, and link within the complex web of pathological events that occur in DN. There are various molecular events that underlie and connect the metabolism, inflammation, and the oxidation in DN. It is known that ROS are molecules that can be beneficial or harmful to the vital processes of redox-sensitive signaling, since the redox state can propagate and adjust the signals from the cellular membrane to the nucleus [39]. Oxidative stress and changes in the AGEs reflect the metabolic and oxidative behaviors that are produced by DM which interfere in multiple signaling pathways [40].

The accumulation of AGEs can be considered as a useful noninvasive marker for tissue damage in DM. A new AGE similar to the 3-deoxyglucosone, methylglyoxal, methionine sulfoxide, and the 2-aminoadipic acid has recently demonstrated having some prognostic power with respect to the progression of DN. However, due to the sophisticated methods required for its detection, it is still far from use in practice. In DN it is frequent to find the conventional markers of oxidative stress in serum, urine, and various organs, among which are the products of lipid peroxidation (4-hydroxynonenal, malondialdehyde) and protein carbonyl groups [32]. There are few valid markers of oxidative stress in the diagnosis and early prognosis of DN. Therefore, deciphering the molecular bases of oxidative stress in DN and other pathologies is required [33]. In an oxidative environment, the polyol pathways promote the generation of AGEs such as N-carboxymethyl-lysine and pentosidine and organize the changes that lead to complications from DM [41]. The AGEs propagate metabolic signals through the interaction of various specific receptors known as RAGEs (the macrophage scavenger receptor and the galectin-3) on inducing the proliferation, apoptosis, autophagy, or migration of the cells, depending on the target cell [42]. The intracellular production of ROS is incited by the AGE-RAGEs interaction [43] through the activation of the peroxisomes proliferator gamma receptor [44]. The transcription factors NFk-B, AP-1, and SP-1 are even further activated by the signaling of the redox-sensitive pathways, which provokes a large quantity of proinflammatory and profibrotic responses. The AGEs and RAGEs are also capable of inducing themselves with the increase in expression of RAGEs, thus augmenting kidney failure. Another factor to consider is age because an increase in age activates cascades, which suggests that the prevention and treatment of DN should be centered not only in the early control of glycemia but also in the limitation of factors related to oxidative stress and the formation of AGEs [45].

## 9. Oxidative Stress in CKD

It is demonstrated that the main cause of morbidity and mortality in patients with CKD is due to CVD and that the oxidative stress together with the subclinical inflammatory state is ultimately responsible for the generation of atherosclerotic plaque [46], since the relationship between oxidative stress and CVD in RRT, with an emphasis on the effects of the loss of antioxidant substances through the filter membranes in hemodialysis or the distinct solutions of peritoneal dialysis, has been demonstrated [47]. In advanced CKD, without exposing the patient to the distinct influences of RRT, oxidation has been demonstrated in patients by what have been called the products of peroxidation: oxidation-reduction glutathione, nuclear and mitochondrial 8-oxo-deoxyguanosine, superoxide dismutase (SOD), glutathione reductase, glutathione peroxidase (GPx), catalase, F2 isoprostanes, and carbonyl proteins. In the present study, all of the markers had significant differences when compared to the control group; the 8-oxo-deoxyguanosine molecule of the nuclear DNA behaved like the most ideal marker to determine oxidative DNA damage. The authors concluded that elevated oxidative stress exists in patients with advanced CKD, which is probably established right from the early stages of the CKD [13].

## 10. Oxidative Stress in Early CKD

Limited information exists on oxidative stress in early CKD [48, 49]; however, the generation of NAPDH oxidase-dependent ROS has been reported as abnormally elevated in patients with CKD in G1 and G2 stages [50]. In these same stages, a decrease in the activity of SOD as well as the GPx, compared to controls, has been reported. Also, the activity of some antioxidants had a negative correlation to the levels of asymmetric dimethylarginine but positive correlation with endothelium-dependent vasodilation of the brachial artery [51].

## 11. Antioxidants

The local and systemic oxidative stress that underlies the pathological characteristics of DN is the result of the imbalance within the production of oxidants/antioxidants, since the oxidative aggression is balanced through powerful antioxidant mechanisms. In uremic patients, there is imbalance with an increase in ROS. The overproduction of ROS induced by hyperglycemia decreases the expression of the manganese superoxide dismutase (mSOD) through the PI3K-AKT-FOXO3 pathway. The mSOD is considered the guardian of the generation of SOD in the mitochondria. The ROS have the ability to produce a decrease in the Sirtuins through modulation of the acetylation of the mitochondrial respiratory chain on stimulating the mitochondrial SOD [52]. Because the ROS are implicated in cellular signaling [53], they are capable of activating the proinflammatory processes and mitogenic cellular pathways that favor the progressive deterioration of kidney function with the appearance of kidney fibrosis and deterioration of the GPx [54], the SOD, catalase, and the nitric oxide synthases (NOS), all of which are considered the most important antioxidant enzymes which detoxify ROS in the kidney [55]. The SOD is the principle physiological defense against oxidative stress which reacts with the superoxide (O_2_^−^) to generate hydrogen peroxide (H_2_O_2_), which is degraded by the catalase and the GPx [56, 57]. The SOD has been demonstrated to suppress albuminuria, levels of TGF-*β*, collagen synthesis, and oxidative stress in experimental models [58]. The GPx uses glutathione to reduce H_2_O_2_ to peroxides of water, acting in conjunction with the peroxynitrite reductase [59].

## 12. Recommended Antioxidants

An extensive review has centered on the study of Curcumin (diferuloylmethane), a component of* Curcuma longa*; in several experimental studies, Curcumin has been demonstrated to modulate multiple molecules of cellular signaling such as the proinflammatory cytokines, apoptosis proteins, transcription factors, TGF-*β*, and diverse endogenous antioxidants [60]. A clinical study performed in 20 patients with type 2 DM and DN demonstrated that short-term supplementation with Curcumin attenuated the proteinuria and the expression of TGF-*β* and interleukin 8. It is important to mention that although Curcumin can be administered as adjuvant therapy, long-term studies with higher numbers of patients are required to confirm the results [61]. In the meta-analysis done by Bjelakovic et al., in 68 randomized trials that included more than 200,000 adults, they compared beta-carotenes, vitamins A, C, and E, and Selenium versus placebo or no intervention. This systematic review showed that only Selenium had the tendency to reduce all of the causes of mortality and that vitamins A and E augmented it, while only vitamin C did not demonstrate any effects [62]. Biesalski et al. reviewed the analysis done by Bjelakovic et al. and concluded that the dietary supplements for the prevention or treatment of chronic illnesses are more effective in patients who ingest insufficient doses, since apparently there is a threshold above which the ingestion of additional nutrients does not offer additional benefits. The threshold for ingestion of nutrients depends on age, gender, health status, and genetic polymorphisms [63].

## 13. Preventive Measures for DN

The early detection and treatment of DN include annual monitoring or more frequently if considered necessary depending on the levels of serum albumin, albuminuria, creatinine in the urine, and the GFR and the improvement and control of glycemia with the objective of achieving a HbA1c < 7% and initiation of ACE inhibitors or ARBs as a first line in managing the illness. It is recommended to initiate therapy with statins in patients <50 years old with concomitant ESRD and DM regardless of the coexistence with DM.

It is imperative to consider that the ESRD will continue augmenting and it is probable that health care systems are not capable of facing the costs. Therefore, the importance of prevention (primary and secondary) to stop the progression of kidney disease and the consequences of ESRD and diminish the associated causes that increase mortality like CVD needs to be emphasized. It is essential to detect patients with ESRD at the onset of clinical or laboratory signs in order to optimize their care and attention [6, 63, 64].

In conclusion, with the early detection of CKD secondary to DN, and with the adequate identification of patients, its impact on health could be diminished and thus, as a result, its impact on society.

## Figures and Tables

**(a):** 

Category of glomerular filtration rate (GFR)		
Category	GFR (mL/min/1.73 m^2^)	Term
G1	≥90	Normal or high
G2	60–89	Slightly decreased
G3a	45–59	Slight or moderately decreased
G3b	30–44	Moderately-to-severely decreased
G4	15–29	Severely decreased
G5	<15	Renal insufficiency

**(b):** 

Category	AER (mg/24 h)	AER (mg/mmol)	AER (mg/g)	Term
A1	<30	<3	<30	Normal to slightly elevated
A2	30–300	3–30	30–300	Moderately elevated
A3	>300	>30	>300	Severely elevated

AER: albumin excretion rate.
